# Heterocyclic Analogs of Thioflavones: Synthesis and NMR Spectroscopic Investigations ^†^

**DOI:** 10.3390/molecules14093814

**Published:** 2009-09-25

**Authors:** Ferdinand C. Fuchs, Gernot A. Eller, Wolfgang Holzer

**Affiliations:** Department of Drug and Natural Product Synthesis, Faculty of Life Sciences, University of Vienna, Althanstrasse 14, A-1090 Vienna, Austria

**Keywords:** thioflavone, Sonogashira coupling, ring closure reactions, NMR spectroscopy

## Abstract

The synthesis of several hitherto unknown heterocyclic ring systems derived from thioflavone is described. Coupling of various *o*-haloheteroarenecarbonyl chlorides with phenylacetylene gives 1-(*o*-haloheteroaryl)-3-phenylprop-2-yn-1-ones, which were treated with NaSH in refluxing ethanol to yield the corresponding bi- and tricyclic annelated 2-phenylthiopyran-4-ones. Detailed NMR spectroscopic investigations of the ring systems and their precursors are presented.

## 1. Introduction

**Figure 1:**
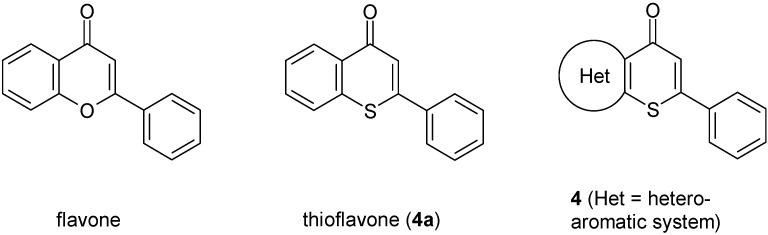
Flavone, thioflavone and its heterocyclic analogs **4**.

The flavone system (2-phenyl-4*H*-chromen-4-one, shown in Figure 1, is the core of many biologically active compounds which play important roles in numerous biological processes. The relevance of flavone-based molecules has been thoroughly described in the literature [1,2,3,4,5]. Replacement of the ring oxygen atom in flavone by a sulfur atom results in thioflavone (**4a**, Figure 1), whose derivatives also exhibit interesting biological properties [6,7,8,9,10,11,12,13]. Moreover, thioflavones are valuable precursors for the synthesis of other condensed heterocyclic systems, such as benzothiazepines [14]. Analogs of type **4** thioflavone-systems – in which the condensed benzene ring of **4a** is replaced by a heteroaromatic moiety (Figure 1) – seem to be of notable interest considering the concept of bioisosterism [15,16,17]. Type **4** compounds with a pyridine, thiophene, benzo[*b*]thiophene or indole system annelated to the thiopyrane ring have been previously characterized.

**Scheme 1:**
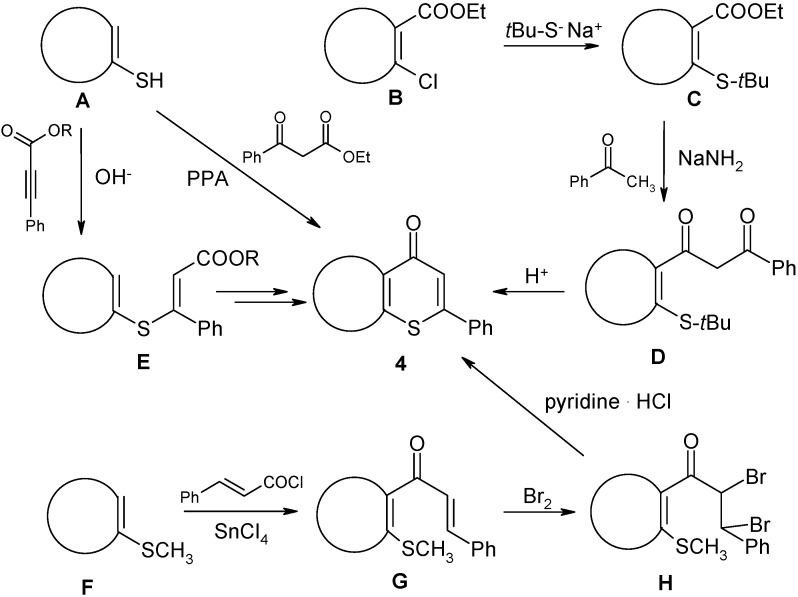
Traditional approaches to the title compounds **4**.

Various approaches have been used to synthesize type **4** systems. Representative examples are given in Scheme 1.

For instance, condensation of appropriate thiophenols **A** with ethyl benzoylacetate followed by subsequent cyclization of the obtained condensation products permits access to type **4** systems [18]. Another approach was employed by Becher in the synthesis of 2-phenyl-4*H*-thiopyrano[2,3-*b*]pyridin-4-one (**4f**) [19]. Ethyl 2-chloronicotinate (**B**) was transformed into the 2-*tert*-butyl congener **C**, which was condensed with acetophenone to yield **D**; the latter readily cyclized into **4f** in an acidic medium. Moreover, the reaction between phenylpropiolates and thiophenols **A** and subsequent ring closure reaction of the formed substituted cinnamates **E** has also been used [20,21], for instance, in the synthesis of indole annelated 2-phenylthiopyran-4-ones [22]. Variants of **4** containing a thiophene or a benzo[*b*]thiophene moiety have been prepared *via* Friedel–Crafts acylation of the corresponding methylthio(benzo)thiophenes **F** with cinnamoyl chloride, to yield ketones **G**, and addition of bromine to the alkene double bond (compounds **H**) and subsequent cyclization with pyridine hydrochloride [23,24,25]. However, many of these methods require precursors that are neither commercially available nor readily synthesized. Moreover, the other above-mentioned approaches lack generality. For example, Friedel-Crafts-based methods are restricted to π-excessive heteroaromatic systems. 

**Scheme 2:**
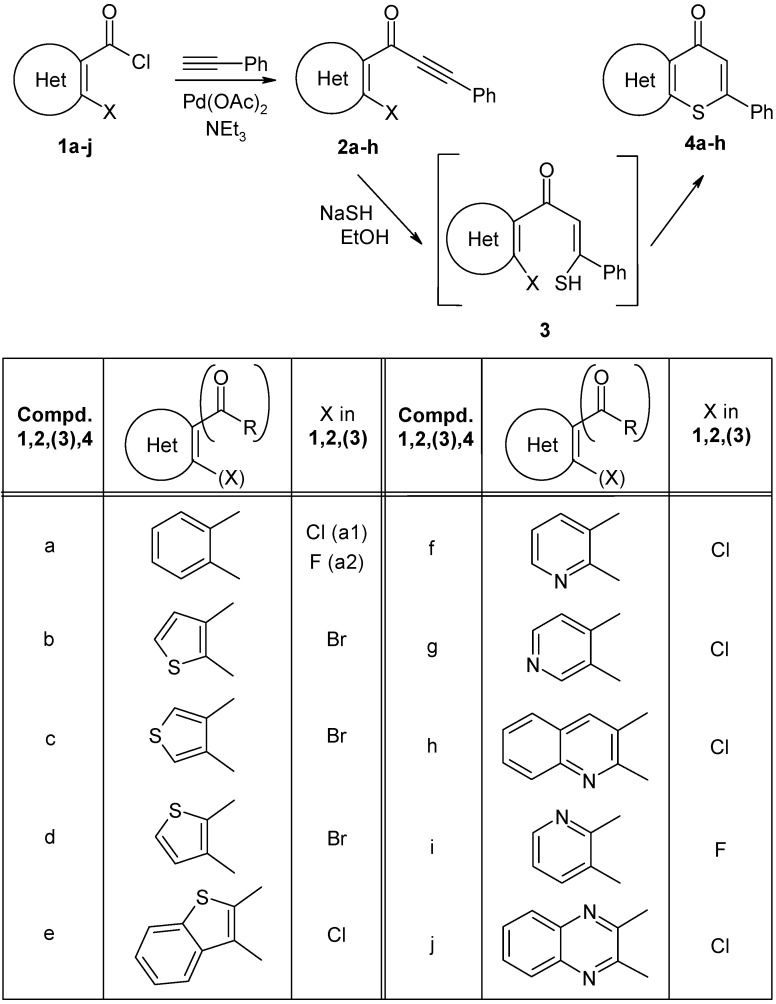
Preparation of compounds **4a-h**.

Here, we present a general approach for synthesizing heterocyclic analogs of type **4** based on a Sonogashira-type coupling of *o*-halo(hetero)aroyl chlorides **1** with phenylacetylene to yield alkynones **2** (Scheme 2). Reactions between **2** and NaSH in refluxing ethanol then yield the desired compounds **4** *via* an addition/cyclization step (Scheme 2). A related approach to thioflavone generation employing a microwave-assisted one-pot, three-component synthesis has been recently described by Müller [26] (based on an earlier report by Shvartsberg [27,28]). In the course of these investigations, the acetylenic component was varied to obtain different 2-substituted 4*H*-thiochromen-4-ones [26].

## 2. Results and Discussion

### 2.1. Chemistry

**Scheme 3:**
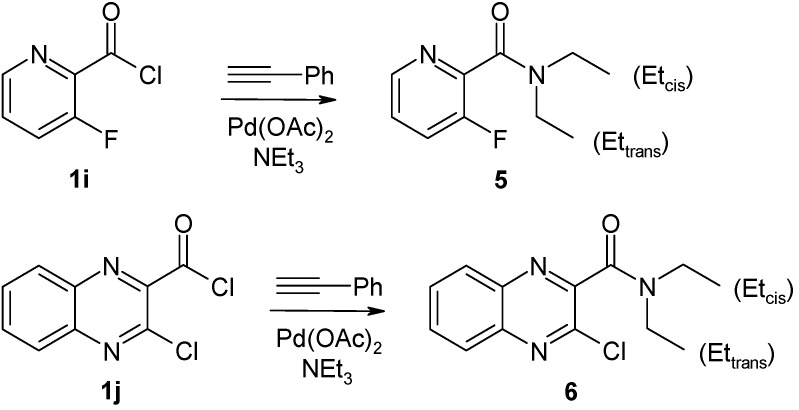
Unexpected formation of *N,N*-diethylamides **5** and **6**.

Synthesis of the target compounds **4** was accomplished *via* the sequence shown in Scheme 2. Precursors **1** are either commercially available or can be easily prepared by treatment of the corresponding carboxylic acids with thionyl chloride. Compounds **1** were transformed into **2** *via* Sonogashira-type coupling [29,30], a very important step in forming C-C bonds with terminal acetylenes [31]. A ligand- and copper-free Pd-catalyzed (Pd acetate, Et_3_N) version of this method for the coupling of carboxylic acid chlorides with different terminal acetylenes has been recently described by Nájera [32] and Srinivasan [33]. In preliminary tests in which the solvent, amount of triethylamine and the acid chloride/phenylacetylene ratio were varied, we adapted the reaction conditions for synthesis of the desired *o*-halo(hetero)arylynones **2a–h**. The best results were obtained using two equivalents of acid chloride, a 3–14-fold molar excess of triethylamine, dichloromethane as the solvent and ambient temperature. Reasonably good yields of ynones **2a–h** were obtained applying these reaction conditions. However, using of **1i** and **1j** as starting materials (both having the COCl group attached in the *o*-position to a pyridine-type nitrogen atom), we did not obtain the corresponding coupling products **2i** or **2j**. Instead, the respective *N,N*-diethylamides **5** and **6** were isolated as the main reaction products from the complex reaction mixtures (Scheme 3). A few reports in the literature have described *N,N*-diethylamide formation from acid chlorides and triethylamine [30,34,35].

**Scheme 4:**
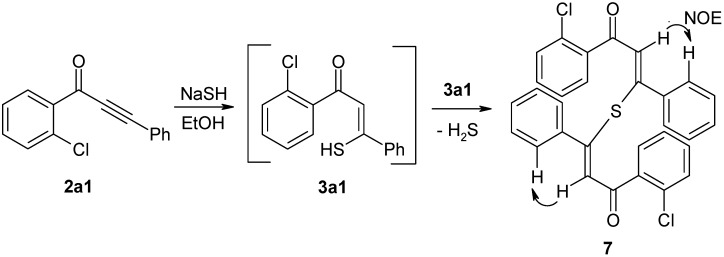
Formation of thioether **7** from Michael addition intermediate **3a1**.

NaSH in refluxing ethanol (96%) was used for the conversion of ynones **2a–h** into thiopyranones **4a–h** (Scheme 2). In principle, it is possible to use Na_2_S as the SH donor [27,28]; however, poor results were obtained using this technique. Evidence of the proposed mechanism, a Michael addition of the hydrosulfide to the alkynone system [26], was obtained in the following experiment. When **2a1** was reacted with NaSH/ethanol at room temperature, we isolated a sulfur-containing product that contained a chlorine atom and an enone group. The high-resolution mass spectrum and the spectral data demonstrated that the product was not the expected intermediate **3a1**, but rather the thioether **7** (Scheme 4), formed following reaction between two units of **3a1** by elimination of hydrogen sulfide. The *cis*-position of alkene-H and the phenyl ring was confirmed by an NOE-difference experiment that employed irradiation of alkene-H resonance (Scheme 4).

Initial attempts to synthesize **4a** and **4f** in a one-pot/two-step procedure (reactants: **1a** or **1f**, respectively, with phenylacetylene, by Pd(OAc)_2_/triethylamine catalysis in solvent; then addition of Na_2_S in DMF) gave low yields (<16%) of the desired thiopyranones.

**Scheme 5:**
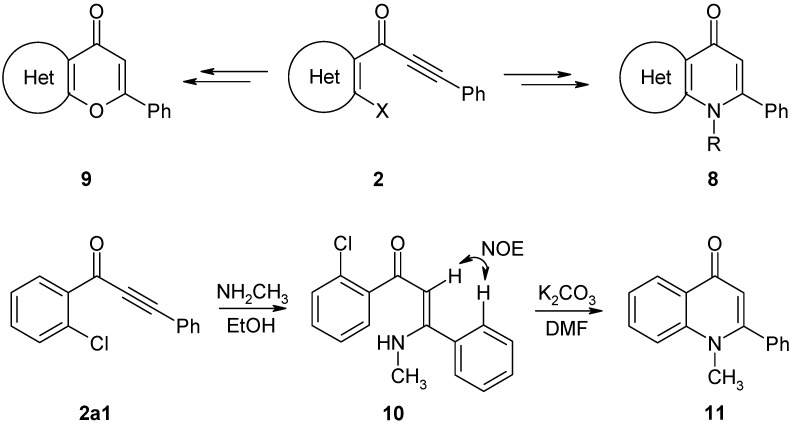
Further synthetic potential of ynones **2**.

The ynones **2** described here are valuable precursors in the synthesis of other condensed heterocyclic systems, such as annelated pyridin-4-ones **8** and pyran-4-ones **9** (Scheme 5). For example, **2a1** was reacted with methylamine to generate the corresponding Michael addition product **10** at a 73% yield (stereochemistry demonstrated by a NOESY experiment). Treatment of product **10** with K_2_CO_3_ in dry DMF gave 1-methyl-2-phenyl-4(1*H*)-quinolinone (**11**) at a good yield. This type of compound was reported to exhibit interesting biological properties, such as anti-platelet [36], anti-mitotic [37], and anti-HIV-replication activity [38]. Furthermore, the well-known class of fluoroquinolinone type [39] antibacterial drugs, such as ciprofloxacin [40] or enoxacin [41], are structurally similar to **11**. Investigations into the preparation of heterocyclic annelated pyridin-4-ones of type **8** are currently in progress and will be reported elsewhere.

### 2.2. Spectroscopic investigations

Alkynones **2** and thiopyranones **4** are predominantly novel structures. Representative structures (**2a1**, **2a2**, **4a**, **4d–f**) have not been thoroughly investigated by spectroscopic methods, such as NMR. In regards to ^13^C-NMR spectroscopy – in cases where such data are available (**4a**, **4f**) – little [19] to no [27,28] signal assignments have been made. Hence, we present the results from an extensive NMR study (^1^H, ^13^C, ^15^N) of compounds **2** and **4**. Reliable and unambiguously assigned chemical shift data are important reference material for NMR prediction programs, such as CSEARCH [42]/NMRPRE-DICT [43] and ACD/C + H predictor [44]. Such programs have become very popular in the last few years, particularly for predicting ^13^C-NMR chemical shifts. However, the quality of such predictions is highly dependent on the availability of authentic reference data from related structures. This criterion is frequently unfulfilled for rare condensed heteroaromatic systems, such as those described here. 

Full and unambiguous assignment of all ^1^H, ^13^C and ^15^N resonances was achieved by combining standard NMR techniques [45], such as fully ^1^H-coupled ^13^C-NMR spectra, APT, HMQC, gs-HSQC, gs-HMBC, COSY, TOCSY, NOESY and NOE-difference spectroscopy. Moreover, experiments with selective excitation (DANTE) of certain ^1^H-resonances were performed, such as long-range INEPT [46] and 2D(δ, *J*) long-range INEPT [47]. The latter experiments were indispensable for the unambiguous mapping of long-range ^13^C,^1^H coupling constants. Aside from the variable heteroaromatic system, the obtained data of the invariant parts of **2** and **4** show a high degree of consistency. Thus, the 3-phenyl-2-propyn-1-one substructure in **2a**–**h** exhibits a carbonyl C-1 shift of 168.3–176.7 ppm, a C-2 shift of 87.2–88.5 ppm, and a C-3 shift of 91.9–96.2 ppm. Alkyne C-atoms, C-2 and C-3, can be easily distinguished by their coupling patterns: the C-2 signal appears as a singlet in the ^1^H-coupled ^13^C-NMR spectrum, whereas the C-3 signal is a triplet due to ^3^*J* coupling with phenyl protons H-2/6 (which is confirmed by a correlation signal from the involved nuclei in the HMBC spectrum). The signals from the phenyl-C atoms are Ph C-1: 119.3–120.1 ppm, Ph C-2/6: 133.1–133.3 ppm, Ph C-3/5: 128.6–128.8 ppm and Ph C-4: 130.9–131.6 ppm. The thiopyranone system in compounds **4a**–**h** is characterized by a chemical shift of 176.2–182.0 ppm for the carbonyl C-atom (split by a ~ 1 Hz ^2^*J* coupling to the adjacent =C-H), 122.1–125.0 ppm for =C-H (^1^*J* 163.3–164.8 Hz) and 151.5–155.1 ppm for S-C-Ph (^2^*J* to =CH ~ 3 Hz). The =C-H proton gives rise to a characteristic singlet signal between 6.99 and 7.32 ppm. As observed with compounds **2**, systems **4** also exhibit small differences in Ph-C shifts within the series **a–h** (Ph C-1: 136.1–136.7 ppm, Ph C-2/6: 126.9–127.2 ppm, Ph C-3/5: 129.2–129.4 ppm, Ph C-4: 130.7–131.3 ppm). The descriptions of NMR spectra in the Experimental section were assigned based on systematic nomenclature and, hence, the numbering of atoms within the thiopyrane moiety is inconsistent.

**Figure 2:**
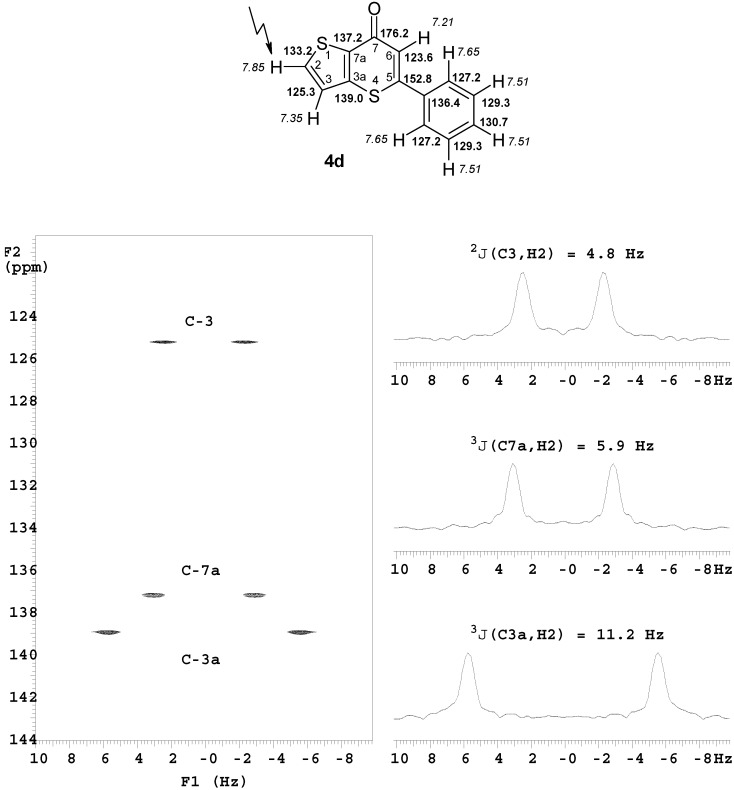
Part of the long-range 2D (δ, *J*) INEPT spectrum of **4d** obtained upon selective excitation of the H-2 resonance; ^1^H- (in *italics*) and ^13^C-NMR chemical shifts in **4d**.

The excellent utility of 2D (*δ*,J) long-range INEPT spectra with selective excitation for the definite mapping of ^13^C,^1^H coupling constants is demonstrated by an example presented in Figure 2. In the ^1^H-coupled ^13^C-NMR spectrum of **4d**, the signal of C-7a is split by a 7.1, 5.9 and 4.7 Hz coupling, whereas coupling occurs with H-2, H-3 and H-6. Unequivocal assignment of these coupling constants based on literature data for the thiophene system is unreliable. However, after selective excitation of the H-2 resonance, the C-7a signal appears as a doublet of 5.9 Hz, thus ^3^*J*(C7a,H2) = 5.9 Hz (Figure 2). Moreover, the couplings ^2^*J*(C3,H2) = 4.8 Hz and ^3^*J*(C3a,H2) = 11.2 Hz emerge (Figure 2) (^4^*J*(C7,H2) = 1.1 Hz also appears, but it is not displayed in Figure 2). Further experiments with selective excitation of H-3 and H-6 assigned ^3^*J*(C7a,H3) = 7.1 Hz and ^2^*J*(C7a,H6) = 4.7 Hz. 

Furthermore, the NMR spectra of *N,N*-diethylcarboxamides **5** and **6**, respectively, two different signals sets for the ethyl moieties (Et_cis_, Et_trans_, see Scheme 3) were found for each case. As expected, this indicates that there is restricted rotation around the amide bond under the recording conditions (CDCl_3_, ambient temperature). 

In the IR spectra, the carbonyl C=O absorption from the ynones **2** appears in the range between 1,601 and 1,649 cm^-1^, and those from the thiopyranones **4** between 1,606 and 1,635 cm^-1^. Absorptions for the C≡C vibration in the IR spectra of compounds **2** appear from 2,196–2,202 cm^-1^.

As reported for related structures [48], the mass spectra of **4** exhibit a fragmentation behavior that is characterized by the loss of CO, a pathway that is also observed for ynones **2**.

## 3. Conclusions

We have presented a widely applicable method for the preparation of heterocyclic annelated 2-phenylthiopyran-4-ones *via* a cross-coupling addition-cyclization approach starting from *o*-halo-heteroaroyl chlorides and phenylacetylene. Detailed NMR spectroscopic studies of the title compounds and their precursors were provided. 

## 4. Experimental

### 4.1. General

Melting points were determined on a Reichert–Kofler hot-stage microscope and are uncorrected. Mass spectra were obtained on a Shimadzu QP 1000 instrument (EI, 70 eV), a Finnigan MAT 8230 instrument (EI, 70 eV, HRMS), and a Finnigan MAT 900S instrument (ESI, 4 kV, MeOH-acetonitrile). IR spectra (KBr unless otherwise stated) were recorded on a Perkin-Elmer FT-IR 1605 spectrophotometer or an ATI Mattson Genesis series FT-IR spectrophotometer. Elemental analyses (C, H, N and S) were performed at the Microanalytical Laboratory, University of Vienna, and were in good agreement (±0.4%) with the calculated values. ^1^H- and ^13^C-NMR spectra were recorded on a Varian UnityPlus 300 spectrometer at 28 °C (299.95 MHz for ^1^H, 75.43 MHz for ^13^C) or on a Bruker Avance 500 spectrometer at 293 K (500.13 MHz for ^1^H, 125.77 MHz for ^13^C). The center of the solvent (CDCl_3_) signal was used as an internal standard, which was related to TMS with δ 7.26 ppm (^1^H) and δ 77.0 ppm (^13^C). ^15^N-NMR spectra (50.68 MHz) and ^19^F-NMR spectra (470.56 MHz) were obtained on a Bruker Avance 500 spectrometer with a ‘directly’ detecting broadband observe probe (BBFO). The spectra were referenced against external nitromethane (^15^N) or against the absolute frequency scale (*Ξ* ratio, ^19^F). The digital resolution was 0.25 Hz/data point in the ^1^H spectra and 0.4 Hz/data point in the ^13^C-NMR spectra. Systematic names were generated with ACD/Name [49] according to the IUPAC recommendations and were checked manually [50]. For chromatographic separations, Kieselgel 60 (70–230 mesh, Merck) was used. Light petroleum had a boiling point of 40–65 °C.

### 4.2. Synthetic procedures

#### 4.2.1. General procedure for the synthesis of *o*-halo(hetero)aroyl chlorides **1b–h**

A suspension of the appropriate acid (2 mmol) in toluene (20 mL), DMF (5 drops) and SOCl_2_ (20 mmol, 2.38 g) was refluxed for 3 h or overnight. The solvent and excess SOCl_2_ were removed under reduced pressure. Additional toluene (4 × 5 mL) was added, and the solvent was removed under reduced pressure. The remaining acid chloride was immediately used in subsequent reaction steps (with no further purification).

#### 4.2.2. General procedure for the synthesis of ynones **2a-h**

The appropriate acid chloride **1** (2 mmol) was dissolved in dry CH_2_Cl_2_ (3 mL). Triethylamine (2 mL), phenylacetylene (1 mmol, 102 mg) and Pd(II) acetate (10 µmol, 2 mg) were added to the solution, which was stirred at room temperature under a N_2_ atmosphere (N_2_ balloon) for the indicated time. The solvent was removed under reduced pressure and water (20 mL) was added to the residue. The resulting solution was acidified with 5% HCl and extracted with CH_2_Cl_2_ or EtOAc (2 × 15 mL). The combined organic layers were washed with a saturated NaHCO_3_ solution and a saturated NaCl solution and were then dried over anhydrous Na_2_SO_4_. After removal of the solvent, the residue was purified by column chromatography through a silica gel column (eluent given below). An analytically pure sample was obtained by recrystallization from the appropriate solvent, which is indicated below.

*1-(2-Chlorophenyl)-3-phenylprop-2-yn-1-one* (**2a1**). Reaction time: 2.5 h. Eluent: toluene/light petroleum, 1:1 v/v. Yield: 209 mg, 87%; orange oil; [51] ^1^H-NMR (300 MHz): δ 8.08 (m, 1H, H-6), 7.65 (m, 2H, Ph H-2,6), 7.48 (m, 2H, H-3,4), 7.46 (m, 1H, Ph H-4), 7.41 (m, 2H, Ph H-3,5), 7.40 (m, 1H, H-5); ^13^C-NMR (75 MHz): δ 176.7 (C=O, ^3^*J*(CO,H6) = 5.3 Hz), 135.9 (C-1), 133.5 (C-2), 133.3 (C-4), 133.1 (Ph C-2,6), 132.4 (C-6), 131.5 (C-3), 130.9 (Ph C-4), 128.7 (Ph C-3,5), 126.8 (C-5), 120.1 (Ph C-1), 93.9 (COC≡C, ^3^*J*(C,Ph H2,6) = 5.5 Hz), 88.3 (COC≡C); IR (liquid film): 2196 (C≡C), 1649 (C=O) cm^−1^; MS *m/z* (%): 242/240 (M^+^, 14/40), 214/212 ([M − C=O]^+^, 25/72) 141/139 ([COC_6_H_4_Cl]^+^, 12/34), 129 ([COC≡CC_6_H_5_]^+^, 100), 101 ([C≡CC_6_H_5_]^+^, 14). 

*1-(2-Fluorophenyl)-3-phenylprop-2-yn-1-one* (**2a2**). Reaction time: 45 min. Eluent: CH_2_Cl_2_/light petroleum, 1:1 v/v. Yield: 164 mg, 73%; light orange oil; [52] ^1^H-NMR (500 MHz): δ 8.11 (m, 1H, H-6), 7.67 (m, 2H, Ph H-2,6), 7.59 (m, 1H, H-4), 7.49 (m, 1H, Ph H-4), 7.42 (m, 2H, Ph H-3,5), 7.28 (m, 1H, H-5), 7.19 (m, 1H, H-3); ^13^C-NMR (125 MHz): δ 174.2 (C=O), 162.1 (C-2, ^1^*J*(C2,F2) = 261.7 Hz), 135.6 (C-4, ^3^*J*(C4,F2) = 9.2 Hz), 133.2 (Ph C-2,6), 131.8 (C-6), 130.9 (Ph C-4), 128.6 (Ph C-3,5), 125.6 (C-1, ^2^*J*(C1,F2) = 7.7 Hz), 124.2 (C-5, ^4^*J*(C5,F2) = 3.9 Hz), 120.1 (Ph C-1), 117.1 (C-3, ^2^*J*(C3,F2) = 21.9 Hz), 93.0 (COC≡C, *J*(C,F2) = 3.2 Hz), 88.5 (COC≡C); ^19^F-NMR (470 MHz): δ −111.3 (F-2); IR (liquid film): 2200 (C≡C), 1647 (C=O) cm^−1^; MS *m/z* (%): 224 (M^+^, 17), 196 ([M − C=O]^+^, 36), 129 ([COC≡CC_6_H_5_]^+^, 100), 123 ([COC_6_H_4_F]^+^, 84), 101 ([C≡CC_6_H_5_]^+^, 21), 74 (83).

*1-(2-Bromothiophen-3-yl)-3-phenylprop-2-yn-1-one* (**2b**). Reaction time: 19 h. Eluent: CH_2_Cl_2_/light petroleum, 3:2 v/v. Yield: 147 mg, 67%; whitish needles, mp 66–67 °C (EtOH/H_2_O); ^1^H- NMR (500 MHz): δ 7.65 (m, 2H, Ph H-2,6), 7.57 (d, ^3^*J* = 5.8 Hz, 1H, H-4), 7.48 (m, 1H, Ph H-4), 7.41 (m, 2H, Ph H-3,5), 7.28 (d, ^3^*J* = 5.8 Hz, 1H, H-5); ^13^C-NMR (125 MHz): δ 170.5 (C=O, ^3^*J*(CO,H4) = 2.0 Hz), 138.2 (C-3, ^2^*J*(C3,H4) = 4.5 Hz, ^3^*J*(C3,H5) = 8.4 Hz), 133.0 (Ph C-2,6), 130.9 (Ph C-4), 129.9 (C-4, ^1^*J*(C4,H4) = 173.4 Hz, ^2^*J*(C4,H5) = 3.8 Hz), 128.7 (Ph C-3,5), 126.0 (C-5, ^1^*J*(C5,H5) = 190.3 Hz, ^2^*J*(C5,H4) = 6.0 Hz), 120.3 (C-2, ^3^*J*(C2,H4) = 12.1 Hz, ^3^*J*(C2,H5) = 6.8 Hz), 120.0 (Ph C-1, ^3^*J*(Ph C1,Ph H3,5) = 8.5 Hz, ^4^*J*(Ph C1,Ph H4) = 1.3 Hz), 93.1 (COC≡C), 88.0 (COC≡C); IR: 2202 (C≡C), 1630 (C=O) cm^−1^; MS *m/z* (%): 292/290 (M^+^, 22/23), 264/262 ([M − C=O]^+^, 28/28), 139 (65), 129 ([COC≡CC_6_H_5_]^+^, 100), 101 ([C≡CC_6_H_5_]^+^, 16), 74 (79). Calcd. for C_13_H_7_BrOS: C, 53.63; H, 2.42; S, 11.01. Found: C, 53.31; H, 2.39; S, 10.92.

*1-(4-Bromothiophen-3-yl)-3-phenylprop-2-yn-1-one* (**2c**). Reaction time: 3 h. Eluent: CH_2_Cl_2_/light petroleum, 3:2 v/v. Yield: 148 mg, 25%; brownish oil; ^1^H-NMR (500 MHz): δ 8.42 (d, ^4^*J* = 3.5 Hz, 1H, H-2), 7.65 (m, 2H, Ph H-2,6), 7.49 (m, 1H, Ph H-4), 7.41 (m, 2H, Ph H-3,5), 7.36 (d, ^4^*J* = 3.5 Hz, 1H, H-5); ^13^C-NMR (125 MHz): δ 170.2 (C=O, ^3^*J*(CO,H2) = 3.6 Hz, ^4^*J*(CO,H5) = 0.6 Hz), 138.6 (C-3, ^2^*J*(C3,H2) = 3.2 Hz, ^3^*J*(C3,H5) = 7.9 Hz), 137.9 (C-2, ^1^*J*(C2,H2) = 189.5 Hz, ^3^*J*(C2,H5) = 5.3 Hz), 133.0 (Ph C-2,6), 130.9 (Ph C-4), 128.7 (Ph C-3,5), 126.1 (C-5, ^1^*J*(C5,H5) = 192.9 Hz, ^3^*J*(C5,H2) = 5.0 Hz), 119.9 (Ph C-1, ^3^*J*(Ph C1,Ph H3,5) = 8.6 Hz, ^4^*J*(Ph C1,Ph H4) = 1.3 Hz), 109.7 (C-4, ^2^*J*(C4,H5) = 0.9 Hz, ^3^*J*(C4,H2) = 11.2 Hz), 91.9 (COC≡C), 87.2 (COC≡C); IR (liquid film): 2189 (C≡C), 1640 (C=O) cm^−1^; MS *m/z* (%): 292/290 (M^+^, 2/2), 264/262 ([M − C=O]^+^, 2/1), 191/189 ([COC_4_H_2_SBr]^+^, 65/61), 129 ([COC≡CC_6_H_5_]^+^, 7), 101 ([C≡CC_6_H_5_]^+^, 1), 81 (100). Calcd. for C_13_H_7_BrOS: C, 53.63; H, 2.42; S, 11.01. Found: C, 53.38; H, 2.50; S, 10.67.

*1-(3-Bromothiophen-2-yl)-3-phenylprop-2-yn-1-one* (**2d**). Reaction time: 23 h. Eluent: CH_2_Cl_2_/light petroleum, 3:2 v/v. Yield: 134 mg, 46%; colorless needles, mp 74–77 °C (EtOH/H_2_O); ^1^H- NMR (500 MHz): δ 7.69 (m, 2H, Ph H2,6), 7.62 (d, ^3^*J* = 5.2 Hz, 1H, H-5), 7.50 (m, 1H, Ph H-4), 7.42 (m, 2H, Ph H-3,5), 7.18 (d, ^3^*J* = 5.2 Hz, 1H, H-4); ^13^C-NMR (125 MHz): δ 168.3 (C=O), 138.0 (C-2, ^3^*J*(C2,H4) = 7.4 Hz, ^3^*J*(C2,H5) = 4.8 Hz), 134.0 (C-4, ^1^*J*(C4,H4) = 176.5 Hz, ^2^*J*(C4,H5) = 4.1 Hz), 133.6 (C-5, ^1^*J*(C5,H5) = 188.1 Hz, ^2^*J*(C5,H4) = 6.1 Hz), 133.1 (Ph C-2,6), 131.0 (Ph C-4), 128.7 (Ph C-3,5), 119.9 (Ph C-1), 116.4 (C-3, ^2^*J*(C3,H4) = 2.4 Hz, ^3^*J*(C3,H5) = 12.2 Hz), 94.1 (COC≡C), 87.3 (COC≡C); IR: 2198 (C≡C), 1627 (C=O) cm^−1^; MS *m/z* (%): 292/290 (M^+^, 25/22), 264/262 ([M − C=O]^+^, 52/47), 139 (95), 129 ([COC≡CC_6_H_5_]^+^, 97), 101 ([C≡CC_6_H_5_]^+^, 22), 84 (73), 74 (77), 43 (100). Calcd. for C_13_H_7_BrOS: C, 53.63; H, 2.42; S, 11.01. Found: C, 53.63; H, 4.00; S, 10.85. HRMS Calcd. for C_13_H_7_BrOS: 289.9401. Found: 289.9403.

*1-(3-Chloro-1-benzo[*b*]thiophen-2-yl)-3-phenylprop-2-yn-1-one* (**2e**). Reaction time: 2.5 h. Eluent: toluene. Yield: 213 mg, 72%; light tan crystals, mp 117–120 °C (MeOH); ^1^H-NMR (500 MHz): δ 8.03 (d, ^3^*J* = 8.0 Hz, 1H, H-4), 7.85 (d, ^3^*J* = 8.1 Hz, 1H, H-7), 7.72 (m, 2H, Ph H-2,6), 7.57 (m, 1H, H-6), 7.52 (m, 2H, H-5, Ph H-4), 7.45 (m, 2H, Ph H-3,5); ^13^C-NMR (125 MHz): δ 169.3 (C=O), 139.3 (C-7a), 137.6 (C-3a), 136.4 (C-2), 133.2 (Ph C-2,6), 131.2 (Ph C-4), 129.0 (C-6), 128.7 (Ph C-3,5), 126.9 (C-3, ^3^*J*(C3,H4) = 7.7 Hz), 125.7 (C-5), 124.4 (C-4), 122.9 (C-7), 119.9 (Ph C-1), 95.1 (COC≡C, ^3^*J*(C,Ph H2,6) = 5.4 Hz), 88.0 (COC≡C); IR: 2199 (C≡C), 1601 (C=O) cm^−1^; MS *m/z* (%): 298/296 (M^+^, 27/68), 270/268 ([M − C=O]^+^, 40/100), 129 ([COC≡CC_6_H_5_]^+^, 100), 101 ([C≡CC_6_H_5_]^+^, 14). Calcd. for C_17_H_9_ClOS•0.2 H_2_O: C, 67.98; H, 3.15. Found: C, 68.11; H, 2.82.

*1-(2-Chloropyridin-3-yl)-3-phenylprop-2-yn-1-one* (**2f**). Reaction time: 2.5 h. Eluent: CH_2_Cl_2_/EtOAc, 20:1 v/v. Yield: 123 mg, 51%; yellowish-brown crystals, mp 69–71 °C (MeOH); ^1^H- NMR (500 MHz): δ 8.56 (dd, ^3^*J*(H5,H6) = 4.7 Hz, ^4^*J* = 1.9 Hz, 1H, H-6), 8.34 (dd, ^3^*J* = 7.7 Hz, ^4^*J* = 1.9 Hz, 1H, H-4), 7.65 (m, 2H, Ph H-2,6), 7.51 (m, 1H, Ph H-4), 7.42 (m, 3H, H-5, Ph H-3,5); ^13^C-NMR (125 MHz): δ 175.5 (C=O, ^3^*J*(CO,H4) = 5.3 Hz), 152.3 (C-6, ^1^*J*(C6,H6) = 183.7 Hz, ^2^*J*(C6,H5) = 3.8 Hz, ^3^*J*(C6,H4) = 8.2 Hz), 149.5 (C-2, ^3^*J*(C2,H4) = 8.8 Hz, ^3^*J*(C2,H6) = 13.8 Hz, ^4^*J*(C2,H5) = 1.5Hz), 140.7 (C-4, ^1^*J*(C4,H4) = 166.2 Hz, ^2^*J*(C4,H5) = 1.9 Hz, ^3^*J*(C4,H6) = 6.7 Hz), 132.6 (C-3), 133.2 (Ph C-2,6), 131.3 (Ph C-4), 128.8 (Ph C-3,5), 122.4 (C-5, ^1^*J*(C5,H5) = 168.2 Hz, ^2^*J*(C5,H6) = 8.2 Hz), 119.5 (Ph C-1, ^3^*J*(Ph C1,Ph H3,5) = 8.6 Hz, ^4^*J*(Ph C1,Ph H4) = 1.4 Hz), 95.7 (COC≡C, ^3^*J*(C,Ph H2,6) = 5.3 Hz), 87.9 (COC≡C); ^15^N-NMR (50 MHz): δ −70.3 (N-1); IR: 2199 (C≡C), 1636 (C=O) cm^−1^; MS *m/z* (%): 243/241 (M^+^, 6/15), 215/213 ([M − C=O]^+^, 6/21), 129 ([COC≡CC_6_H_5_]^+^, 100), 101 ([C≡CC_6_H_5_]^+^, 13). Calcd. for C_14_H_8_ClNO: C, 69.58; H, 3.34; N, 5.80. Found: C, 69.59; H, 3.16; N, 5.67.

*1-(3-Chloropyridin-4-yl)-3-phenylprop-2-yn-1-one* (**2g**). Reaction time: 23 h. Eluent: CH_2_Cl_2_/EtOAc, 20:1 v/v. Yield: 70 mg, 19%; brown crystals, mp 77–79 °C (MeOH/H_2_O, 5:1 v/v); ^1^H- NMR (500 MHz): δ 8.76 (s, 1H, H-2), 8.68 (d, ^3^*J* = 4.9 Hz, 1H, H-6), 7.81 (d, ^3^*J* = 4.9 Hz, 1H, H-5), 7.65 (m, 2H, Ph H-2,6), 7.52 (m, 1H, Ph H-4), 7.43 (m, 2H, Ph H-3,5); ^13^C-NMR (125 MHz): δ 175.3 (C=O), 151.6 (C-2, ^1^*J*(C2,H2) = 188.0 Hz, ^3^*J*(C2,H6) = 11.4 Hz, ^4^*J*(C2,H5) = 1.0 Hz), 148.4 (C-6, ^1^*J*(C6,H6) = 183.6 Hz, ^2^*J*(C6,H5) = 2.3 Hz, ^3^*J*(C6,H2) = 11.6 Hz), 141.8 (C-4, ^2^*J*(C4,H5) = not resolved, ^3^*J*(C4,H2) = 4.9 Hz, ^3^*J*(C4,H6) = 6.9 Hz), 133.3 (Ph C-2,6), 131.6 (Ph C-4), 129.3 (C-3), 128.8 (Ph C-3,5), 124.0 (C-5, ^1^*J*(C5,H5) = 167.1 Hz, ^2^*J*(C5,H6) = 9.7 Hz), 119.3 (Ph C-1, ^3^*J*(Ph C1,Ph H3,5) = 8.5 Hz, ^4^*J*(Ph C1,Ph H4) = 1.4 Hz), 96.2 (COC≡C, ^3^*J*(C,Ph H2,6) = 5.4 Hz), 87.8 (COC≡C); IR: 2197 (C≡C), 1641 (C=O) cm^−1^; MS *m/z* (%): 243/241 (M^+^, 4/12), 215/213 ([M − C=O]^+^, 4/8), 129 ([COC≡CC_6_H_5_]^+^, 100), 101 ([C≡CC_6_H_5_]^+^, 11). Calcd. for C_14_H_8_ClNO•0.2 H_2_O: C, 68.56; H, 3.45; N, 5.71. Found: C, 68.69; H, 3.25; N, 5.63.

*1-(2-Chloroquinolin-3-yl)-3-phenylprop-2-yn-1-one* (**2h**). Reaction time: 200 min. Eluent: light petroleum/EtOAc, 4:1 v/v. Yield: 181 mg, 62%; brownish-yellow crystals, mp 100–101 °C (MeOH); ^1^H-NMR (500 MHz): δ 8.87 (s, 1H, H-4), 8.08 (dd, ^3^*J* = 8.5 Hz, ^4^*J* = 1.1 Hz, 1H, H-8), 7.97 (dd, ^3^*J* = 8.1 Hz, ^4^*J* = 1.4 Hz, 1H, H-5), 7.88 (m, ^3^*J*(H7,H6) = 7.0 Hz, ^3^*J*(H7,H8) = 8.5 Hz, ^4^*J* = 1.4 Hz, 1H, H-7), 7.68 (m, 2H, Ph H-2,6), 7.66 (m, 1H, H-6), 7.51 (m, 1H, Ph H-4), 7.44 (m, 2H, Ph H-3,5); ^13^C-NMR (125 MHz): δ 175.3 (C=O, ^3^*J*(CO,H4) = 5.7 Hz), 148.4 (C-8a), 147.0 (C-2, ^3^*J*(C2,H4) = 9.7 Hz), 142.7 (C-4, ^1^*J*(C4,H4) = 164.4 Hz, ^3^*J*(C4,H5) = 4.6 Hz), 133.2 (C-7, Ph C-2,6), 131.3 (Ph C-4), 130.2 (C-3), 128.9 (C-5), 128.8 (Ph C-3,5), 128.5 (C-8), 128.0 (C-6), 126.0 (C-4a), 119.7 (Ph C-1, ^3^*J*(Ph C1,Ph H3,5) = 8.5 Hz, ^4^*J*(Ph C1,Ph H4) = 1.3 Hz), 95.2 (COC≡C, ^3^*J*(C,Ph H2,6) = 5.3 Hz, ^4^*J*(C,Ph H3,5) = 1.0 Hz), 88.0 (COC≡C); ^15^N-NMR (50 MHz): δ −78.4 (N-1); IR: 2197 (C≡C), 1632 (C=O) cm^−1^; MS *m/z* (%): 293/291 (M^+^, 6/15), 265/263 ([M − C=O]^+^, 8/24), 239 (41), 129 ([COC≡CC_6_H_5_]^+^, 90), 101 ([C≡CC_6_H_5_]^+^, 45), 43 (100). Calcd. for C_18_H_10_ClNO•0.4 H_2_O: C, 72.32; H, 3.64; N, 4.69. Found: C, 72.27; H, 3.37; N, 5.01.

#### 4.2.3. General procedure for the synthesis of thiopyranones **4a–h**

A suspension of NaSH hydrate (~60%, 1.2 mmol, 112 mg) was refluxed in EtOH (20 mL) until a cloudy solution was formed. Ynone **2** (0.4 mmol) in EtOH (2 mL) was then added to the solution, which was refluxed for the indicated time. The solvent was removed under reduced pressure and water (20 mL) was added to the residue. The resulting solution was acidified with 5% HCl and extracted with CH_2_Cl_2_ (2 × 15 mL). The combined organic layers were washed with a saturated NaHCO_3_ solution and a saturated NaCl solution. They were then dried over anhydrous Na_2_SO_4_. After removal of the solvent, the residue was purified by column chromatography through a silica gel (eluent as indicated below). An analytically pure sample was obtained by recrystallization from an appropriate solvent.

*2-Phenyl-4*H*-thiochromen-4-one* (**4a**). Reaction time: 4 h. Eluent: CH_2_Cl_2_/EtOAc, 15:1 v/v. Yield: 131 mg, 54%; beige needles, mp 121–123 °C (MeOH) (lit. [18] mp 124–126 °C); ^1^H= NMR (500 MHz): δ 8.54 (dd, 1H, H-5), 7.68 (m, 2H, Ph H-2,6), 7.65 (dd, 1H, H-8), 7.62 (dt, 1H, H-7), 7.54 (dt, 1H, H-6), 7.51 (m, 1H, Ph H-4), 7.50 (m, 2H, Ph H-3,5), 7.24 (s, 1H, H-3); ^13^C-NMR (125 MHz): δ 180.8 (C-4), 153.0 (C-2), 137.6 (C-8a), 136.5 (Ph C-1), 131.6 (C-7), 130.8 (C-4a, Ph C-4), 129.2 (Ph C-3,5), 128.5 (C-5), 127.7 (C-6), 126.9 (Ph C-2,6), 126.4 (C-8), 123.4 (C-3, ^1^*J*(C3,H3) = 163.6 Hz); IR: 1620 (C=O) cm^−1^; MS *m/z* (%): 238 (M^+^, 92), 210 ([M − C=O]^+^, 100), 136 ([COC_6_H_4_S]^+^, 53), 108 ([C_6_H_4_S]^+^, 28).

*6-Phenyl-4*H*-thieno[2,3-*b*]thiopyran-4-one* (**4b**). Reaction time: 100 min. Eluent: CH_2_Cl_2_/EtOAc, 5:1 v/v. Yield: 20 mg, 50%; whitish powder, mp 115–117 °C (MeOH); ^1^H-NMR (500 MHz): δ 7.78 (d, ^3^*J* = 5.3 Hz, 1H, H-3), 7.62 (m, 2H, Ph H-2,6), 7.50 (m, 1H, Ph H-4), 7.49 (m, 2H, Ph H-3,5), 7.46 (d, ^3^*J* = 5.3 Hz, 1H, H-2), 7.20 (s, 1H, H-5); ^13^C-NMR (125 MHz): δ 177.1 (C-4, ^2^*J*(C4,H5) = 1.2 Hz, ^3^*J*(C4,H3) = 1.3 Hz), 151.5 (C-6, ^2^*J*(C6,H5) = 3.4 Hz), 144.3 (C-7a, ^3^*J*(C7a,H2) = 6.7 Hz, ^3^*J*(C7a,H3) = 10.0 Hz), 137.5 (C-3a, ^2^*J*(C3a,H3) = 4.7 Hz, ^3^*J*(C3a,H2) = 9.9 Hz, ^3^*J*(C3a,H5) = 3.7 Hz), 136.1 (Ph C-1, ^3^*J*(Ph C1,H5) = 5.4 Hz), 130.7 (Ph C-4), 129.3 (Ph C-3,5), 127.0 (Ph C-2,6), 125.5 (C-3, ^1^*J*(C3,H3) = 174.8 Hz, ^2^*J*(C3,H2) = 4.4 Hz, ^4^*J*(C3,H5) = 1.0 Hz), 125.3 (C-2, ^1^*J*(C2,H2) = 189.6 Hz, ^2^*J*(C2,H3) = 7.5 Hz), 125.0 (C-5, ^1^*J*(C5,H5) = 163.3 Hz); IR: 1606 (C=O) cm^−1^; MS *m/z* (%): 244 (M^+^, 100), 216 ([M − C=O]^+^, 31), 142 ([COC_4_H_2_S_2_]^+^, 97), 120 ([COCH=CC_6_H_5_]^+^, 69), 114 ([C_4_H_2_S_2_]^+^, 38), 101 ([C=CC_6_H_5_]^+^, 27). Calcd. for C_13_H_8_OS_2_: C, 63.90; H, 3.30. Found: C, 63.57; H, 3.27.

*2-Phenyl-4*H*-thieno[3,4-*b*]thiopyran-4-one* (**4c**). Reaction time: 22.5 h. Eluent: CH_2_Cl_2_/EtOAc, 5:1 v/v. Yield: 28 mg, 34%; brownish needles, mp 160–162 °C (MeOH/H_2_O); ^1^H-NMR (500 MHz): δ 8.51 (d, ^4^*J* = 3.4 Hz, 1H, H-5), 7.66 (m, 2H, Ph H-2,6), 7.52 (d, ^3^*J* = 3.4 Hz, 1H, H-7), 7.49 (m, 1H, Ph H-4), 7.48 (m, 2H, Ph H-3,5), 6.99 (s, 1H, H-3); ^13^C-NMR (125 MHz): δ 178.2 (C-4, ^2^*J*(C4,H3) = 1.1 Hz, ^3^*J*(C4,H5) = 2.2 Hz, ^4^*J*(C4,H7) = 1.1 Hz), 153.3 (C-2, ^2^*J*(C2,H3) = 3.1 Hz), 136.7 (Ph C-1, ^3^*J*(Ph C1,H3) = 5.5 Hz), 134.7 (C-4a, ^2^*J*(C4a,H5) = 3.2 Hz, ^3^*J*(C4a,H3) = 3.8 Hz, ^3^*J*(C4a,H7) = 7.8 Hz), 131.4 (C-7a, ^2^*J*(C7a,H7) = 2.8 Hz, ^3^*J*(C7a,H5) = 9.8 Hz), 130.8 (Ph C-4), 129.7 (C-5, ^1^*J*(C5,H5) = 191.8 Hz, ^3^*J*(C5,H7) = 4.9 Hz, ^4^*J*(C5,H3) = 1.3 Hz), 129.2 (Ph C-3,5), 127.0 (Ph C-2,6), 122.1 (C-3, ^1^*J*(C3,H3) = 163.6 Hz), 119.2 (C-7, ^1^*J*(C7,H7) = 189.3 Hz, ^3^*J*(C7,H5) = 5.7 Hz); IR: 1612 (C=O) cm^−1^; MS *m/z* (%): 244 (M^+^, 48), 216 ([M − C=O]^+^, 16), 142 ([COC_4_H_2_S_2_]^+^, 100), 114 ([C_4_H_2_S_2_]^+^, 36), 82 ([C_4_H_2_S]^+^, 22). Calcd. for C_13_H_8_OS_2_: C, 63.90; H, 3.30. Found: C, 62.82; H 3.28. HRMS (ESI) Calcd. for C_13_H_9_OS_2_ [M+H]: 245.0095. Found: 245.0100.

*5-Phenyl-7*H*-thieno[3,2-*b*]thiopyran-7-one* (**4d**). Reaction time: 1 h. Eluent: CH_2_Cl_2_/EtOAc, 5:1 v/v. Yield: 80 mg, 87%; brownish powder, mp 82–84 °C (EtOH/H_2_O) (lit. [24] mp 80 °C); ^1^H-NMR (500 MHz): δ 7.85 (d, ^3^*J* = 5.3 Hz, 1H, H-2), 7.65 (m, 2H, Ph H-2,6), 7.51 (m, 3H, Ph H-3,4,5), 7.35 (d, ^3^*J* = 5.3 Hz, 1H, H-3), 7.21 (s, 1H, H-6); ^13^C-NMR (125 MHz): δ 176.2 (C-7, ^2^*J*(C7,H6) = 1.1 Hz, ^4^*J*(C7,H2) = 1.1 Hz, ^4^*J*(C7,H3) = 1.1 Hz), 152.8 (C-5, ^2^*J*(C5,H6) = 3.1 Hz), 139.0 (C-3a, ^2^*J*(C3a,H3) = 4.4 Hz, ^3^*J*(C3a,H2) = 11.2 Hz), 137.2 (C-7a, ^3^*J*(C7a,H2) = 5.9 Hz, ^3^*J*(C7a,H3) = 7.1 Hz, ^3^*J*(C7a,H6) = 4.7 Hz), 136.4 (Ph C-1, ^3^*J*(Ph C1,H6) = 5.4 Hz), 133.2 (C-2, ^1^*J*(C2,H2) = 186.6 Hz, ^2^*J*(C2,H3) = 6.0 Hz), 130.7 (Ph C-4), 129.3 (Ph C-3,5), 127.2 (Ph C-2,6), 125.3 (C-3, ^1^*J*(C3,H3) = 173.4 Hz, ^2^*J*(C3,H2) = 4.8 Hz), 123.6 (C-6, ^1^*J*(C6,H6) = 163.9 Hz); MS *m/z* (%): 244 (M^+^, 46), 243 ([M − H]^+^, 21), 216 ([M − C=O]^+^, 16), 142 ([COC_4_H_2_S_2_]^+^, 94), 114 ([C_4_H_2_S_2_]^+^, 33), 102 ([CH=CC_6_H_5_]^+^, 19), 82 ([C_4_H_2_S]^+^, 20), 69 (100).

*2-Phenyl-4*H*-thiopyrano[3,2-*b*][1]benzothiophen-4-one* (**4e**). Reaction time: 70 min. Eluent: CH_2_Cl_2_/EtOAc, 15:1 v/v. Yield: 96 mg, 82%; colorless crystals, mp 166–167 °C (MeOH/EtOAc, 2:1 v/v) (lit. [25] mp 177 °C); ^1^H-NMR (500 MHz): δ 8.00 (m, 1H, H-9), 7.99 (m, 1H, H-6), 7.71 (m, 2H, Ph H-2,6), 7.59 (m, 1H, H-7), 7.54 (m, 3H, Ph H-3,4,5), 7.53 (m, 1H, H-8), 7.32 (s, 1H, H-3); ^13^C-NMR (125 MHz): δ 177.0 (C-4, ^2^*J*(C4,H3) = 1.1 Hz), 152.1 (C-2, ^2^*J*(C2,H3) = 3.1 Hz), 140.7 (C-5a), 137.3 (C-4a, ^3^*J*(C4a,H3) = 4.6 Hz), 136.3 (Ph C-1, ^3^*J*(Ph C1,H3) = 5.3 Hz), 136.2 (C-9a), 135.4 (C-9b, ^3^*J*(C9b,H9) = 3.9 Hz), 130.8 (Ph C-4), 129.4 (Ph C-3,5), 128.6 (C-7, ^1^*J*(C7,H7) = 162.0 Hz, ^3^*J*(C7,H9) = 7.8 Hz), 127.2 (Ph C-2,6), 125.3 (C-8, ^1^*J*(C8,H8) = 162.5 Hz, ^3^*J*(C8,H6) = 7.6 Hz), 124.7 (C-3, ^1^*J*(C3,H3) = 163.9 Hz), 123.8 (C-6, ^1^*J*(C6,H6) = 164.8 Hz, ^2^*J*(C6,H7) = 1.5 Hz, ^3^*J*(C6,H8) = 8.0 Hz, ^4^*J*(C6,H9) = 1.5 Hz), 122.5 (C-9, ^1^*J*(C9,H9) = 161.1 Hz, ^2^*J*(C9,H8) = 1.4 Hz, ^3^*J*(C9,H7) = 8.1 Hz, ^4^*J*(C9,H6) = 1.4 Hz); IR: 1618 (C=O) cm^−1^; MS *m/z* (%): 294 (M^+^, 32), 266 ([M–C=O]^+^, 34), 164 ([C_8_H_4_S_2_]^+^, 25), 132 ([C_8_H_4_S]^+^, 29), 120 (43), 68 (100). Calcd. for C_17_H_10_OS_2_•0.1 H_2_O: C, 68.94; H, 3.47; S, 21.65. Found: C, 68.91; H, 3.45; S, 21.26.

*2-Phenyl-4*H*-thiopyrano[2,3-*b*]pyridin-4-one* (**4f**). Reaction time: 1.5 h. Eluent: CH_2_Cl_2_/EtOAc, 15:1 v/v. Yield: 36 mg, 93%; luminous yellow needles, mp 114–117 °C (MeOH) (lit. [19] mp 113–116 °C); ^1^H-NMR (300 MHz): δ 8.81 (dd, ^3^*J* = 4.5 Hz, ^4^*J* = 1.9 Hz, 1H, H-7), 8.77 (dd, ^3^*J* = 8.1 Hz, ^4^*J* = 1.9 Hz, 1H, H-5), 7.71 (m, 2H, Ph H-2,6), 7.53 (m, 3H, Ph H-3,4,5), 7.50 (dd, ^3^*J*(H6,H7) = 4.5 Hz, ^3^*J*(H6,H5) = 8.1 Hz, 1H, H-6), 7.26 (s, 1H, H-3); ^13^C-NMR (75 MHz): δ 181.3 (C-4, ^3^*J*(C4,H5) = 3.5 Hz), 159.1 (C-8a, ^3^*J*(C8a,H5) = 6.5 Hz, ^3^*J*(C8a,H7) = 14.0 Hz), 154.7 (C-2), 152.8 (C-7, ^1^*J*(C7,H7) = 181.7 Hz, ^2^*J*(C7,H6) = 3.8 Hz, ^3^*J*(C7,H5) = 7.8 Hz), 136.7 (C-5, ^1^*J*(C5,H5) = 168.2 Hz, ^2^*J*(C5,H6) not resolved, ^3^*J*(C5,H7) = 6.2 Hz), 136.3 (Ph C-1), 131.1 (Ph C-4), 129.4 (Ph C-3,5), 128.1 (C-4a), 127.0 (Ph C-2,6), 123.6 (C-3, ^1^*J*(C3,H3) = 164.7 Hz), 122.9 (C-6, ^1^*J*(C6,H6) = 167.0 Hz, ^2^*J*(C6,H5) not resolved, ^2^*J*(C6,H7) = 8.2 Hz); ^15^N-NMR (50 MHz): δ −76.8 (N-8); IR: 1630 (C=O) cm^−1^; MS *m/z* (%): 239 (M^+^, 100), 211 ([M − C=O]^+^, 99), 137 ([COC_5_H_3_NS]^+^, 33), 109 ([C_5_H_3_NS]^+^, 57), 105 ([COC_5_H_3_N]^+^, 32), 102 ([CH=CC_6_H_5_]^+^, 33). Calcd. for C_14_H_9_NOS: C, 70.27; H, 3.79; N, 5.85. Found: C, 70.26; H, 4.04; N, 5.75.

*2-Phenyl-4*H*-thiopyran[2,3-*c*]pyridin-4-one* (**4g**). Reaction time: 1.5 h. Eluent: CH_2_Cl_2_/EtOAc, 5:1 v/v. Yield: 21 mg, 44%; flat brownish prisms, mp 153–154 °C (EtOH/H_2_O); ^1^H-NMR (500 MHz): δ 9.02 (s, 1H, H-8), 8.73 (d, ^3^*J* = 5.3 Hz, 1H, H-6), 8.27 (d, ^3^*J* = 5.3 Hz, 1H, H-5), 7.69 (m, 2H, Ph H-2,6), 7.53 (m, 1H, Ph H-4), 7.52 (m, 2H, Ph H-3,5), 7.27 (s, 1H, H-3); ^13^C-NMR (125 MHz): δ 179.5 (C-4, ^2^*J*(C4,H3) = 1.0 Hz, ^3^*J*(C4,H5) = 3.6 Hz, ^4^*J*(C4,H8) = 1.6 Hz), 154.0 (C-2, ^2^*J*(C2,H3) = 2.5 Hz), 148.8 (C-8, ^1^*J*(C8,H8) = 183.4 Hz, ^3^*J*(C8,H6) = 11.1 Hz, ^4^*J*(C8,H5) = 0.8 Hz), 147.8 (C-6, ^1^*J*(C6,H6) = 182.7 Hz, ^2^*J*(C6,H5) = 3.2 Hz, ^3^*J*(C6,H8) = 11.6 Hz), 136.1 (Ph C-1, ^3^*J*(Ph C1,H3) = 5.5 Hz), 135.7 (C-4a, ^2^*J*(C4a,H5) not resolved, ^3^*J*(C4a,H3) = 3.7 Hz, ^3^*J*(C4a,H6) = 6.7 Hz, ^3^*J*(C4a,H8) = 5.1 Hz), 133.2 (C-8a, ^2^*J*(C8a,H8) = 7.9 Hz, ^3^*J*(C8a,H5) = 6.5 Hz, ^4^*J*(C8a,H6) = 1.8 Hz), 131.3 (Ph C-4), 129.4 (Ph C-3,5), 127.0 (Ph C-2,6), 123.9 (C-3, ^1^*J*(C3,H3) = 164.5 Hz), 120.6 (C-5, ^1^*J*(C5,H5) = 168.9 Hz, ^2^*J*(C5,H6) = 9.0 Hz, ^4^*J*(C5,H3) = 0.8 Hz, ^4^*J*(C5,H8) = 1.7 Hz); ^15^N-NMR (50 MHz): δ −60.0 (N-7); IR: 1635 (C=O) cm^−1^; MS *m/z* (%): 239 (M^+^, 100), 211 ([M − C=O]^+^, 77), 149 (49), 139 (28), 137 ([COC_5_H_3_NS]^+^, 66), 109 ([C_5_H_3_NS]^+^, 53), 105 ([COC_5_H_3_N]^+^, 20), 102 ([CH=CC_6_H_5_]^+^, 41), 81 (98). Calcd. for C_14_H_9_NOS: C, 70.27; H, 3.79; N, 5.85; S, 13.40. Found: C, 70.28; H, 4.02; N, 5.69; S, 13.21. HRMS Calcd. for C_14_H_9_NOS: 239.0405. Found: 239.0402.

*2-Phenyl-4*H*-thiopyrano[2,3-*b*]quinolin-4-one* (**4h**). Reaction time: 100 min. Eluent: CH_2_Cl_2_/EtOAc, 20:1 v/v. Yield: 77 mg, 67%; off-white needles, mp 208–210 °C (CH_2_Cl_2_/EtOAc, 5:1 v/v); ^1^H-NMR (500 MHz): δ 9.30 (s, 1H, H-5), 8.09 (d, ^3^*J* = 8.6 Hz, 1H, H-9), 8.04 (d, ^3^*J* = 8.1 Hz, 1H, H-6), 7.88 (m, 1H, H-8), 7.74 (m, 2H, Ph H-2,6), 7.62 (m, 1H, H-7), 7.54 (m, 3H, Ph H-3,4,5), 7.23 (s, 1H, H-3); ^13^C-NMR (125 MHz): δ 182.0 (C-4, ^2^*J*(C4,H3) = 0.7 Hz, ^3^*J*(C4,H5) = 4.1 Hz), 157.1 (C-10a, ^3^*J*(C10a,H5) = 8.1 Hz), 155.1 (C-2), 149.2 (C-9a), 138.6 (C-5, ^1^*J*(C5,H5) = 166.4 Hz, ^3^*J*(C5,H6) = 4.7 Hz), 136.4 (Ph C-1), 133.1 (C-8), 131.2 (Ph C-4), 129.7 (C-6), 129.4 (Ph C-3,5), 128.1 (C-9), 127.2 (C-7), 127.0 (Ph C-2,6), 126.9 (C-5a), 125.1 (C-4a), 122.1 (C-3, ^1^*J*(C3,H3) = 164.8 Hz); ^15^N-NMR (50 MHz): δ −83.8 (N-10); IR: 1631 (C=O) cm^−1^; MS *m/z* (%): 289 (M^+^, 91), 261 ([M − C=O]^+^, 88), 181 (46), 159 ([C_9_H_5_NS]^+^, 39), 130 ([COCH=CC_6_H_5_]^+^, 77), 101 ([C=CC_6_H_5_]^+^, 44), 75 (82), 68 (100). Calcd. for C_18_H_11_NOS•0.2 H_2_O: C, 73.80; H, 3.92; N, 4.78. Found: C, 73.85; H, 3.71; N, 4.77.

#### 4.2.4. Preparation of *N,N*-diethyl-3-fluoropyridine-2-carboxamide (**5**)

Under the conditions given for the synthesis of compounds **2**, reaction of **1i** (2 mmol, 319 mg) with phenylacetylene (1 mmol, 102 mg), Et_3_N (2 mL) and Pd(II) acetate (10 µmol, 2 mg) for 26 h resulted in formation of compound **5** after purification by column chromatography (eluent: EtOAc). Yield: 131 mg, 33%; brown oil; ^1^H-NMR (500 MHz): δ 8.42 (dd, ^3^*J* = 4.6 Hz,^ 4^*J* = 1.1 Hz, ^5^*J*(H6,F3) = 1.4 Hz, 1H, H-6), 7.47 (dd, ^3^*J*(H4, H5*)* = 8.5 Hz, ^3^*J*(H4,F3) = 8.9 Hz, ^4^*J* = 1.1 Hz, 1H, H-4), 7.35 (m, ^3^*J*(H5,H6) = 4.6 Hz, ^3^*J*(H5,H4) = 8.5 Hz, ^4^*J*(H5,F3) = 4.2 Hz, 1H, H-5), 3.58 (q, ^3^*J* = 7.1 Hz, 2H, N-CH_2_ cis), 3.20 (q, ^3^*J* = 7.1 Hz, 2H, N-CH_2_ trans), 1.27 (t, ^3^*J* = 7.1 Hz, 3H, CH_3_ cis), 1.10 (t, ^3^*J* = 7.1 Hz, 3H, CH_3_ trans); ^13^C-NMR (125 MHz): δ 164.7 (C=O, ^3^*J*(CO,F3) = 3.1 Hz), 155.8 (C-3, ^1^*J*(C3,F3) = 259.1 Hz, ^2^*J*(C3,H4) = 4.8 Hz, ^3^*J*(C3,H5) = 9.6 Hz, ^4^*J*(C3,H6) = 1.9 Hz), 145.2 (C-6, ^1^*J*(C6,H6) = 182.3 Hz, ^2^*J*(C6,H5) = 2.7 Hz, ^3^*J*(C6,H4) = 7.3 Hz, ^4^*J*(C6,F3) = 5.0 Hz), 144.0 (C-2, ^2^*J*(C2,F3) = 18.0 Hz, ^3^*J*(C2,H4) = 3.8 Hz, ^3^*J*(C2,H6) = 12.2 Hz, ^4^*J*(C2,H5) = 1.5 Hz), 125.1 (C-5, ^1^*J*(C5,H5) = 167.2 Hz, ^2^*J*(C5,H4) = 2.0 Hz, ^2^*J*(C5,H6) = 9.4 Hz, ^3^*J*(C5,F3) = 3.8 Hz), 123.9 (C-4, ^1^*J*(C4,H4) = 167.4 Hz, ^2^*J*(C4,F3) = 18.4 Hz, ^3^*J*(C4,H6) = 7.2 Hz), 42.8 (N-CH_2_ trans, ^1^*J* = 135.2 Hz, ^2^*J*(CH_2_,CH_3_) = 4.2 Hz), 39.4 (N-CH_2_ cis, ^1^*J* = 136.5 Hz, ^2^*J*(CH_2_,CH_3_) = 4.2 Hz), 14.0 (CH_3_ trans, ^1^*J* = 127.1 Hz, ^2^*J*(CH_3_,CH_2_) = 3.1 Hz), 12.8 (CH_3_ cis, ^1^*J* = 127.2 Hz, ^2^*J*(CH_3_,CH_2_) = 3.4 Hz); ^15^N-NMR (50 MHz): δ −65.8 (N-1), −248.9 (CONR_2_); ^19^F-NMR (470 MHz): δ −124.3 (F-3, ^3^*J*(F3,H4) = 8.9 Hz, ^4^*J*(F3,H5) = 4.2 Hz, ^5^*J*(F3,H6) = 1.4 Hz); IR: 1643 (C=O) cm^−1^; MS *m/z* (%): 196 (M^+^, 7), 124 ([M − N(Et)_2_]^+^, 65), 97 ([C_5_H_3_NF]^+^, 55), 72 ([N(Et)_2_]^+^, 100), 44 (63). Calcd. for C_10_H_13_FN_2_O•0.2 H_2_O: C, 60.11; H, 6.76; N, 14.02. Found: C, 60.28; H, 6.59; N, 13.90.

#### 4.2.5. Preparation of 3-chloro-*N,N*-diethylquinoxaline-2-carboxamide (**6**)

Under the conditions given for synthesis of compounds **2**, reaction of **1j** (2 mmol, 455 mg) with phenylacetylene (1 mmol, 102 mg), Et_3_N (2 mL) and Pd(II) acetate (10 µmol, 2 mg) for 20 h resulted in formation of compound **6** after purification by column chromatography (eluent: CH_2_Cl_2_/EtOAc, 5:1 v/v.). Yield: 256 mg, 49%; brownish needles, mp 91–93 °C (MeOH); ^1^H-NMR (500 MHz): δ 8.10 (m, 1H, H-8), 8.03 (m, 1H, H-5), 7.83 (m, 1H, H-6), 7.80 (m, 1H, H-7), 3.65 (q, ^3^*J* = 7.2 Hz, 2H, N-CH_2_ cis), 3.20 (q, ^3^*J* = 7.1 Hz, 2H, N-CH_2_ trans), 1.34 (t, ^3^*J* = 7.2 Hz, 3H, CH_3_ cis), 1.16 (t, ^3^*J* = 7.1 Hz, 3H, CH_3_ trans); ^13^C-NMR (125 MHz): δ 164.8 (C=O), 149.1 (C-3), 143.8 (C-2), 141.6 (C-4a), 140.0 (C-8a), 131.6 (C-6), 130.8 (C-7), 129.2 (C-8), 128.3 (C-5), 42.9 (N-CH_2_ trans), 39.5 (N-CH_2_ cis), 13.8 (CH_3_ trans), 12.5 (CH_3_ cis); ^15^N-NMR (50 MHz): δ –52.1 (N-1), −63.0 (N-4), −249.2 (CONR_2_); IR: 1635 (C=O) cm^−1^; MS *m/z* (%): 265/263 (M^+^, 0.5/1.5), 191 ([M − N(Et)_2_]^+^, 3), 165/163 ([C_8_H_4_N_2_Cl]^+^, 7/18), 128 ([C_8_H_4_N_2_]^+^, 5), 72 ([N(Et)_2_]^+^, 100). Calcd. for C_13_H_14_ClN_3_O: C, 59.21; H, 5.35; N, 15.93. Found: C, 59.43; H, 5.42; N, 15.99.

#### 4.2.6. Preparation of (2*Z*,2'*Z*)-3,3'-sulfanediylbis[1-(2-chlorophenyl)-3-phenylprop-2-en-1-one] (**7**)

Ynone **2a1** (0.5 mmol, 120 mg) in EtOH (2 mL) was added to a suspension of NaSH hydrate (~60%, 1.3 mmol, 121 mg) in EtOH (20 mL). The resulting solution was stirred at room temperature for 30 min, and then processed as described in the preparation of compounds **4**. The residue was purified by column chromatography through silica gel (eluent: CH_2_Cl_2_). Yield: 48 mg, 19%; yellow powder, mp 161–164 °C (EtOH); ^1^H-NMR (300 MHz): δ 7.65 (m, 2H, H-6), 7.43 (m, 2H, H-3), 7.42 (m, 2H, H-4), 7.38 (m, 2H, H-5), 7.18 (m, 2H, Ph H-4), 7.05 (m, 4H, Ph H-3,5), 6.97 (m, 4H, Ph H-2,6), 6.92 (s, 2H, COCH=C); ^13^C-NMR (75 MHz): δ 189.7 (C=O), 155.4 (COCH=C), 140.6 (Ph C-1), 139.7 (C-1), 131.9 (C-4), 131.4 (C-2), 130.5 (C-6), 130.2 (C-3), 128.9 (Ph C-4), 128.3 (Ph C-2,6), 128.0 (Ph C-3,5), 127.7 (COCH=C, ^1^*J* = 161.5 Hz), 127.1 (C-5); IR: 1638 (C=O) cm^−1^; MS *m/z* (%): 516/514 (M^+^, 0.3/0.4), 275/273 ([M − C_15_H_10_ClO]^+^, 13/33), 238 (19), 149 ([COC(S)C_6_H_5_]^+^, 80), 141/139 ([COC_6_H_4_Cl]^+^, 37/100), 113/111 ([C_6_H_4_Cl]^+^, 29/49), 105 (38), 85 ([COCH=CS]^+^, 32), 77 ([C_6_H_5_]^+^, 33), 57 (83). HRMS (ESI) Calcd. for C_30_H_21_Cl_2_O_2_S [M + H]: 515.0639. Found: 515.0642.

#### 4.2.7. Preparation of (2*Z*)-1-(2-chlorophenyl)-3-(methylamino)-3-phenylprop-2-en-1-one (**10**)

Ethanolic methylamine-solution (33 wt%, 6 mL, 48 mmol) was added to a solution of ynone **2a1** (0.5 mmol, 120 mg) in pyridine (0.4 mL) and H_2_O (3 drops). The solution was refluxed for 1 h. The solvent was then removed under reduced pressure and water (20 mL) was added to the residue. The resulting solution was adjusted to pH 5 with 5% HCl and extracted with CH_2_Cl_2_ (2 × 15 mL). The combined organic layers were washed with a saturated NaHCO_3_ solution and a saturated NaCl solution and then dried over anhydrous Na_2_SO_4_. After removal of the solvent, the residue was purified by column chromatography through silica gel (eluent: CH_2_Cl_2_/EtOAc, 20:1 v/v.), which produced **10** as a slowly crystallizing oil. Yield: 99 mg, 73%; pale orange solid, mp 77–80 °C; ^1^H-NMR (500 MHz): δ 11.18 (broad, 1H, N-H), 7.49 (m, 1H, H-6), 7.44 (m, 3H, Ph H-3,4,5), 7.42 (m, 2H, Ph H-2,6), 7.37 (m, 1H, H-3), 7.27 (m, 1H, H-4), 7.26 (m, 1H, H-5), 5.44 (s, 1H, COCH=C), 2.96 (d, ^3^*J* = 5.4 Hz, 3H, N-CH_3_);^13^C-NMR (125 MHz): δ 189.2 (C=O), 167.6 (COCH=C), 141.3 (C-1), 134.8 (Ph C-1), 130.8 (C-2), 130.1 (C-3), 129.9 (C-4), 129.7 (Ph C-4), 129.2 (C-6), 128.5 (Ph C-3,5), 127.7 (Ph C-2,6), 126.5 (C-5), 97.3 (COCH=C, ^1^*J* = 164.5 Hz, ^3^*J*(C,NH) = 3.6 Hz), 31.6 (N-CH_3_, ^1^*J* = 138.5 Hz, ^2^*J*(NCH_3_,NH) = 3.4 Hz, ^4^*J*(NCH_3_,COCH=C) = 1.1 Hz); ^15^N-NMR (50 MHz): δ −281.5 (N-CH_3_); IR: 1595 (C=O) cm^−1^; MS *m/z* (%): 273/271 (M^+^, 12/43), 256/254 (22/45), 236 ([M − Cl]^+^, 100), 160 ([M–C_6_H_4_Cl]^+^, 57), 141/139 ([COC_6_H_4_Cl]^+^, 14/33), 117 ([CH=C(NH)C_6_H_5_]^+^, 66), 113/111 ([C_6_H_4_Cl]^+^, 13/38), 104 ([HNCC_6_H_5_]^+^, 25), 102 ([CH=CC_6_H_5_]^+^, 97), 77 ([C_6_H_5_]^+^, 74). Calcd. for C_16_H_14_ClNO: C, 70.72; H, 5.19; N, 5.15. Found: C, 70.32; H, 5.22; N, 5.32.

#### 4.2.8. Preparation of 1-methyl-2-phenylquinolin-4(1*H*)-one (**11**)

Potassium carbonate (2 mmol, 277 mg) was added to a solution of **10** (220 µmol, 60 mg) in DMF (5 mL). The resulting mixture was refluxed for 52 h under a N_2_ atmosphere (N_2_ balloon). The solvent was then removed under reduced pressure and water (20 mL) was added to the residue. The resulting solution was adjusted to a slightly basic pH with 5% HCl and extracted with CH_2_Cl_2_ (2 ° 15 mL). The combined organic layers were washed with a saturated NaHCO_3_ solution and a saturated NaCl solution and then dried over anhydrous Na_2_SO_4_. After removal of the solvent, the oily residue was purified by column chromatography through silica gel (eluent: EtOAc/NEt_3_, 20:1 v/v.) to generate **11**. Yield: 37 mg, 72%; yellowish crystals, mp 137–140 °C (EtOH/H_2_O) (lit. [53] mp 142–145 °C); ^1^H-NMR (500 MHz): δ 8.50 (dd, ^3^*J* = 8.1 Hz, ^4^*J* = 1.7 Hz, 1H, H-5), 7.71 (ddd, ^3^*J*(H7,H6) = 7.0 Hz, ^3^*J*(H7,H8) = 8.6 Hz, ^4^*J* = 1.7 Hz, 1H, H-7), 7.55 (d, ^3^*J* = 8.6 Hz, 1H, H-8), 7.50 (m, 3H, Ph H-3,4,5), 7.42 (m, ^3^*J*(H6,H7) = 7.0 Hz, ^3^*J*(H6,H5) = 8.1 Hz, ^4^*J* = 1.0 Hz, 1H, H-6), 7.41 (m, 2H, Ph H-2,6), 6.29 (s, 1H, H-3), 3.60 (s, 3H, N-CH_3_); ^13^C-NMR (125 MHz): δ 177.6 (C-4, ^2^*J*(C4,H3) = 1.4 Hz, ^3^*J*(C4,H5) = 3.9 Hz), 154.7 (C-2, ^2^*J*(C2,H3) = 3.7 Hz, ^3^*J*(C2,NCH_3_) = 3.1 Hz), 141.9 (C-8a, ^3^*J*(C8a,H5) = 7.6 Hz, ^3^*J*(C8a,H7) = 9.5 Hz, ^3^*J*(C8a,NCH_3_) = 2.9 Hz), 135.9 (Ph C-1, ^3^*J*(Ph C1,H3) = 3.9 Hz), 132.3 (C-7), 129.6 (Ph C-4), 128.8 (Ph C-3,5), 128.5 (Ph C-2,6), 126.8 (C-4a, ^3^*J*(C4a,H3) = 4.8 Hz), 126.7 (C-5, ^3^*J*(C5,H7) = 7.8 Hz), 123.6 (C-6), 115.9 (C-8), 112.7 (C-3, ^1^*J*(C3,H3) = 165.8 Hz), 37.2 (N-CH_3_, ^1^*J* = 140.1 Hz); ^15^N-NMR (50 MHz): δ −262.1 (N-1); MS *m/z* (%): 235 (M^+^, 100), 207 ([M–C=O]^+^, 100), 206 ([M–H,–C=O]^+^, 55), 102 (40), 77 ([C_6_H_5_]^+^, 47).

## References

[1] Harborne J.B. (1994). The Flavonoids – Advances in Research Since 1986.

[2] Anderson O.M., Markham K.R. (2006). Flavonoids: Chemistry, Biochemistry and Applications.

[3] Birt D.F., Hendrich S., Wang W. (2001). Dietary agents in cancer prevention: Flavonoids and isoflavonoids. Pharmacol. Ther..

[4] Kim H.P., Son K.H., Chang H.W., Kang S.S. (2004). Anti-inflammatory Plant Flavonoids and Cellular Action Mechanisms. J. Pharmacol. Sci..

[5] Seelinger G., Merfort I., Schempp C.M. (2008). Anti-oxidant, anti-inflammatory and anti-allergic activities of luteolin. Planta Med..

[6] Nakazumi H., Ueyama T., Kitao T. (1984). Synthesis and antibacterial activity of 2-phenyl-4*H*-benzo[*b*]thiopyran-4-ones (Thioflavones) and related Compounds. J. Heterocycl. Chem..

[7] Nakazumi H., Ueyama T., Kitao T. (1985). Antimicrobial activity of 3-(substituted methyl)-2-phenyl-4*H*-1-benzothiopyran-4-ones. J. Heterocycl. Chem..

[8] Nakazumi H., Kobara Y., Kitao T. (1992). Synthesis and insecticidal activity of 4-(aminomethyl)-2*H*-1-benzothiopyran-2-ones (thiocoumarins) and related compounds. J. Heterocycl. Chem..

[9] Razdan R.K., Bruni R.J., Mehta A.C., Weinhardt K.K., Papanastassiou Z.B. (1978). A new class of antimalarial drugs: derivatives of benzothiopyrans. J. Med. Chem..

[10] Ares J.J., Outt P.E., Randall J.L., Johnston J.N., Murray P.D., O’Brien L.M., Weisshaar P.S., Ems B.L. (1996). Synthesis and biological evaluation of flavonoids and related compounds as gastroprotective agents. Bioorg. Med. Chem. Lett..

[11] Dhanak D., Keenan R.M., Burton G., Kaura A., Darcy M.G., Shah D.H., Ridgers L.H., Breen A., Lavery P., Tew D.G., West A. (1998). Benzothiopyran-4-one based reversible inhibitors of the human cytomegalovirus (HCMV) protease. Bioorg. Med. Chem. Lett..

[12] Wang H.-K., Bastow K.F., Cosentino L.M., Lee K.-H. (1996). Antitumor Agents. 166. Synthesis and biological evaluation of 5,6,7,8-substituted-2-phenylthiochromen-4-ones. J. Med. Chem..

[13] Dekermendjian K., Kahnberg P., Witt M.-R., Sterner O., Nielsen M., Liljefors T. (1999). Structure-activity relationships and molecular modeling analysis of flavonoids binding to the benzodiazepine site of the rat brain gaba_a_ receptor complex. J. Med. Chem..

[14] Dike S.Y., Ner D.H., Kumar A. (1991). A New chemoenzymatic enantioselective synthesis of optically active benzothiopyran and benzothiazepin ring system. Synlett.

[15] Burger A. (1991). Isosterism and bioisosterism in drug design. Progr. Drug Res..

[16] Patani G.A., LaVoie E.J. (1996). Bioisosterism: A rational approach in drug design. Chem. Rev..

[17] Lima L.M., Barreiro E.J. (2005). Bioisosterism: A Useful strategy for molecular modification and drug design. Curr. Med. Chem..

[18] Bossert F. (1964). Über eine neue thiochromon-synthese. Justus Liebigs Ann. Chem..

[19] Becher J., Christensen M.C., Møller J., Winckelmann I. (1982). The Synthesis of 4*H*-thiopyrano[2,3-*b*]pyridin-4-ones. Sulfur Lett..

[20] Wadsworth D.H., Detty M.R. (1980). Regiochemical control of the addition of aryl selenols and aryl thiols to the triple bond of arylpropiolates. Synthesis of seleno- and thioflavones and seleno- and thioaurones. J. Org. Chem..

[21] Taylor A.W., Dean D.K. (1988). A new synthesis of thioflavones. Tetrahedron Lett..

[22] Brown R.E. (1976). Indolothiopyrones. U.S. Patent.

[23] Henrio G., Morel J. (1974). First synthesis of flavone type compounds containing a thiophen ring. Tetrahedron Lett..

[24] Henrio G., Morel J., Pastour P. (1977). Sur la synthèse d’isostères thiophèniques des flavones, thioflavones et xanthones. Tetrahedron.

[25] Netchitailo P., Decroix B., Morel J., Pastour P. (1978). Synthèse de flavones et de xanthones dans la série du benzo[4,5]thiophène. J. Heterocycl. Chem..

[26] Willy B., Müller T.J.J. (2009). A novel consecutive three-component Coupling-Addition-S_N_Ar (CASNAR) synthesis of 4*H*-thiochromen-4-ones. Synlett.

[27] Shvartsberg M.S., Ivanchikova I.D. (2003). Synthesis of sulfur-containing heterocyclic compounds by cyclo-condensation of acetylenic derivatives of anthraquinone with sodium sulfide. ARKIVOC.

[28] Ivanchikova I.D., Shvartsberg M.S. (2004). Synthesis of anthrathiopyrantriones by heterocyclization of alkynoyl derivatives of chloroanthraquinones. Russ.Chem. Bull..

[29] Sonogashira K., Tohda Y., Hagihara N. (1975). A convenient synthesis of acetylenes: Catalytic substitutions of acetylenic hydrogen with bromoalkenes, iodoarenes, and bromopyridines. Tetrahedron Lett..

[30] Tohda Y., Sonogashira K., Hagihara N. (1977). A Convenient synthesis of 1-alkynyl ketones and 2-alkynamides. Synthesis.

[31] Chinchilla R., Nájera C. (2007). The Sonogashira reaction: A booming methodology in sythetic organic chemistry. Chem. Rev..

[32] Alonso D.A., Nájera C., Pacheco M.C. (2004). Synthesis of ynones by palladium-catalyzed acylation of terminal alkynes with acid chlorides. J. Org. Chem..

[33] Palimkar S.S., Kumar P.H., Jogdand N.R., Daniel T., Lahoti R.J., Srinivasan K.V. (2006). Copper-, ligand- and solvent-free synthesis of ynones by coupling acid chlorides with terminal alkynes. Tetrahedron Lett..

[34] Chowdhury C., Kundu N.G. (1999). Studies on copper(I) catalysed cross-coupling reactions: A convenient and facile method for the synthesis of diversely substituted α,β-acetylenic ketones. Tetrahedron.

[35] Cox R.J., Ritson D.J., Dane T.A., Berge J., Charmant J.P.H., Kantacha A. (2005). Room temperature palladium catalysed coupling of acyl chlorides with terminal alkynes. Chem. Commun..

[36] Huang L.-J., Hsieh M.-C., Teng C.-M., Lee K.-H., Kuo S.-C. (1998). Synthesis and Antiplatelet Activity of Phenyl Quinolones. Bioorg. Med. Chem..

[37] Kuo S.-C., Lee H.-Z., Juang J.-P., Lin Y.-T., Wu T.-S., Chang J.-J., Lednicer D., Paull K.D., Lin C.M., Hamel E., Lee K.-H. (1993). Synthesis and Cytotoxicity of 1,6,7,8-substituted 2-(4’-Substituted phenyl)-4-quinolones and related compounds: Identification as antimitotic agents interacting with tubulin. J. Med. Chem..

[38] Gatto B., Tabarrini O., Massari S., Giaretta G., Sabatini S., Del Vecchio C., Parolin C., Fravolini A., Palumbo M., Cecchetti V. (2009). 2-phenylquinolones as inhibitors of the HIV-1 Tat-TAR interaction. ChemMedChem..

[39] Hooper D.C., Rubinstein E. (2003). Quinolone Antimicrobial Agents.

[40] Kleemann A., Engel J. (1999). Pharmaceutical Substances.

[41] Kleemann A., Engel J. (1999). Pharmaceutical Substances.

[42] Kalchhauser H., Robien W. (1985). CSEARCH: A computer program for identification of organic compounds and fully automated assignment of carbon-13 nuclear magnetic resonance spectra. J. Chem. Inf. Comput. Sci..

[43] (2004). NMR Predict, version 3.2.8.

[44] (2006). ACD/C+H Predictors and DB, version 10.04.

[45] Braun S., Kalinowski H.-O., Berger S. (1998). 150 and More Basic NMR Experiments.

[46] Bax A. (1984). Structure determination and spectral assignment by pulsed polarization transfer via long-range proton-carbon-13 couplings. J. Magn. Reson..

[47] Jippo T., Kamo O., Nagayama N. (1986). Determination of long-range proton-carbon 13 coupling constants with selective two-dimensional INEPT. J. Magn. Reson..

[48] Kuhn F., Oehme M., Romero F., Abou-Mansour E., Tabacchi R. (2003). Differentiation of isomeric flavon/isoflavone aglycones by MS^2^ ion trap mass spectrometry and a double neutral loss of CO. Rapid Commun. Mass Spectrom..

[49] (2006). ACD Name, version 10.01.

[50] Eller G.A. (2006). Improving the quality of published chemical names with nomenclature software. Molecules.

[51] Laliberte R., Campbell D.J., Bruderlein F. (1967). Antihelmintic activities of chalcones and related compounds. Can. J. Pharm. Sci..

[52] Utekhina N.V., Surov Yu.N., Korzhova N.V., Kazantseva V.M., Orlov V.D., Korshunov S.P. (1987). Basicity and polarity of aromatic α,β-acetylenic ketones. Zhur. Obsh. Khim..

[53] Coppola G.M. (1982). The chemistry of 2*H*-3,1-benzoxazine-2,4(1*H*)-dione (Isatoic Anhydride). 9. synthesis of 2-arylquinoline alkaloids. J. Heterocycl. Chem..

